# OCT4: A penetrant pluripotency inducer

**DOI:** 10.1186/2045-9769-3-6

**Published:** 2014-03-31

**Authors:** Xuecong Wang, Ralf Jauch

**Affiliations:** Guangzhou Institutes of Biomedicine and Health, Chinese Academy of Sciences (GIBH), 190 Kai Yuan Avenue, Science Park, Guangzhou, 510530 China

**Keywords:** OCT4, Cell penetrating peptide, Induced pluripotent stem cells, Reprogramming, Pluripotency

## Abstract

Native OCT4 protein has the intrinsic ability of crossing cellular membranes to enter cells. This finding could revive efforts to induce pluripotency with proteins replacing nucleic acid-based approaches, and raises the intriguing question as to whether OCT4 can act non-cell-autonomously.

## Commentary

The octamer binding protein 4 (OCT4) is one of the prominent transcription factor proteins that featured in Yamanaka’s original four factor-cocktail (OCT4, SOX2, KLF4 and c-MYC). This combination of transcription factors is capable of inducing the reprogramming of somatic cells into induced pluripotent stem cells (iPSCs) in mouse and human [1]. OCT4 is also present in many alternative cocktails achieving a similar feat [2] and, when brought into certain cell types, is able to induce pluripotency alone without the help of additional factors [3]. Initially, reprogramming factors were introduced into cells using retroviral vectors that integrate into the genome with the side effect of causing potentially harmful mutations to the host [4]. Clearly, such genetic alterations should be avoided if cells derived from such procedures are to be used in clinical applications. Consequently, various studies have focused on the derivation of iPSCs that obviate genomic integration. For example, episomal vectors containing six reprogramming factors (OCT4, SOX2, NANOG, LIN28, KLF4 and c-MYC) are now commonly used [5]. Likewise, adenoviral vectors [6, 7] and non-integrating DNA based plasmids [8, 9] were explored as non-integrating reprogramming strategies. Moreover, the use of modified RNAs constituted a further step towards the generation of safe and genetically unscathed iPSCs [10]. However, at least some of the nucleic acid based reprogramming strategies carry residual risks of modifying the host’s genome, also present problems with tightly balanced dosing and the exposure time cannot easily be controlled [4]. Collectively, those caveats could hamper regulatory approval of therapeutic cells derived from nucleic acid based reprogramming strategies. The search for alternative iPSC generation strategies that avoid nucleic acids altogether has therefore continued. The next logical step after using DNA and RNA based delivery of reprogramming factors was the usage of cell penetrating versions of the four proteins themselves. Indeed, protein-induced pluripotent stem cells (piPSCs) could be generated by using recombinant proteins expressed in *E. coli* supplemented with valproic acid [11], recombinant proteins from crude HEK293 cell extract [12] or from total embryonic stem cell (ESC) extract [13]. piPSCs generation normally relies on tags to facilitate cell permeation such as poly-arginine (9 arginines [12] or 11 arginines [11]) or an equally highly basic 12 amino acids peptide derived from the human immunodeficiency virus type 1 (HIV-1) Tat protein (TAT) [14]. However, since proteins derived from crude cellular extracts of ESCs could support piPSCs formation, the addition of such fusion tags may not be essential [13]. Disappointingly though, the efficiency of protein induced reprogramming is very low and, therefore, piPSCs have not become popular until recently. In this regard, the discovery that the activation of innate immunity could profoundly improve the production of piPSCs could be a game changer and push the approach to center stage [15].

The findings reported by Harreither et al. in a recent issue of Cell Regeneration [16] could further boost the popularity of piPSCs. Rather than attaching a cell penetrating peptide (CPP) tag that was previously deemed necessary when piPSCs were generated with recombinant proteins, the authors wondered if the OCT4 protein could enter cells without modifications. Several other proteins are known to have the intrinsic ability to penetrate the membrane barriers of the cell. Amongst them is the well-known CPP penetratin derived from the homeodomain of Antennapedia. Coincidentally, OCT4 contains a bi-partite POU domain to bind DNA consisting of a POU specific and a POU homeodomain. Harreither et al. realized that a 16-amino-acid peptide derived from the third helix of the homeodomain of OCT4 has 68% amino acid similarity with penetratin and hypothesized that it would translocate into living cells thereby functioning as a CPP. Indeed, the OCT4 peptide N-terminally labeled with fluorescein isothiocyanate (FITC) could enter cells within 1 hour, suggesting uptake efficiency even higher than that of the penetratin control. Further experiments suggested that the penetration occurs via the endocytic pathway. Encouragingly, the OCT4-CPP did not appear to get stuck in endosomes but was found homogenously throughout the cytoplasm and even within the nucleus. Moreover, the OCT4-CPP can be used as vehicle to support the translocation of otherwise non-penetrating cargo proteins. For example, OCT4-CPP-Cre fusion protein could readily enter CVI-5B cells containing a loxP-modified reporter system. However, the CPP activity was found to be weaker than other typical CPPs such as TAT. The next obvious question was whether the new CPP could support cellular entry of the unmodified full length OCT4 protein. To test this, the authors used human OCT4 purified from *E. coli* inclusion bodies and incubated it with CVI-5B cells and human BJ foreskin fibroblasts at a concentration of 100 nM. Immunostaining revealed that the OCT4 protein penetrated both cell types, suggesting that the unmodified OCT4 protein can be used as self-penetrating pluripotency reprogramming factor without the addition of cationic fusion tag.

**Figure 1:**
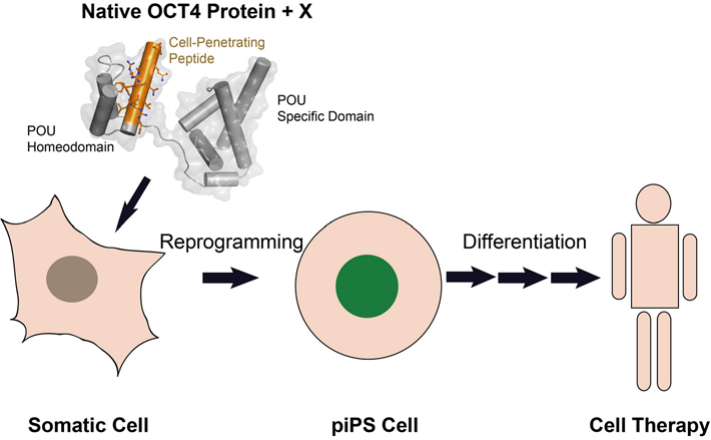
**The third helix of the human OCT4 protein contains a cell penetrating peptide (orange) enabling the native protein to enter somatic cells.** Potentially, native (or truncated) OCT4 can be used to generate integration-free piPSCs in combination with other factors such as small molecules [22] or other cell-penetrating factors and activators of the innate immunity [15] (indicated as ‘X’ in the figure) that can serve as raw materials to produce clinical grade cells for safe cell therapy.

The use of recombinant proteins for cellular reprogramming would eliminate the risks of nucleic acid based approaches and could prove to be a versatile way to generate iPSCs. Notably, a truncated version of the transcription factor Nanog consisting of only the 70 amino acid homedomain retains the capacity to promote reprogramming [17]. Similarly, versions of Sox proteins trimmed to their DNA binding high mobility group (HMG) domain still support reprogramming when a VP16 transactivation domain is added [18]. Collectively, one might thus envisage cocktails of reprogramming factors, truncated and/or enhanced with transactivation domains and CPPs [19], that readily cross cellular membranes and promote reprogramming (Figure 1). Previously, poor delivery, the cumbersome production of recombinant proteins and low reprogramming efficiency has hampered the widespread use of piPSCs. Yet, self-penetrating proteins (e.g. OCT4) and truncated proteins might be more readily available and unleash a renaissance of the piPSCs technology. Clearly, proteins could allow for more accurate dosing, highly defined timing of factor exposure and maximal control over the sequence of factor addition not easily possible with other techniques. Controllability is very desirable, since both the efficiency [20] as well as the outcome [21] strongly depends on exposure time and the sequence of factor addition. The recently reported production of chemically induced pluripotent stem cells (CiPSCs) that relied solely on a combination of 7 small-molecule compounds [22] is another promising alternative to standard reprogramming approaches, but still awaits widespread acceptance in the field.

Besides its practical implications for reprogramming purposes, the present work also raises some intriguing mechanistic questions. Some homeodomain transcription factors were found to jump from cell to cell on an organismic level. For example, Otx2 can function non-cell-autonomously by transferring from the retina to the visual cortex where it contributes to neurophysiological responses triggered by visual experiences [23]. Is OCT4 also able to execute gene expression programs in blastocyst cells where its gene is actually silenced because the OCT4 protein is taken up by a paracrine signaling mechanism? If true, this would imply that cell penetration is a two-way street and OCT4 is secreted and taken up with similar efficiency.

In summary, the study by Harreither et al. emphasizes that piPSCs are still in the race. To date, the jury is still out on which approach will be most effective, tunable and safe to produce clinical grade iPSCs.

## References

[1] Takahashi K, Tanabe K, Ohnuki M, Narita M, Ichisaka T, Tomoda K, Yamanaka S (2007). Induction of pluripotent stem cells from adult human fibroblasts by defined factors. Cell.

[2] Yu J, Vodyanik MA, Smuga-Otto K, Antosiewicz-Bourget J, Frane JL, Tian S, Nie J, Jonsdottir GA, Ruotti V, Stewart R, Slukvin II, Thomson JA (2007). Induced pluripotent stem cell lines derived from human somatic cells. Science.

[3] Kim JB, Greber B, Arauzo-Bravo MJ, Meyer J, Park KI, Zaehres H, Scholer HR (2009). Direct reprogramming of human neural stem cells by OCT4. Nature.

[4] Robinton DA, Daley GQ (2012). The promise of induced pluripotent stem cells in research and therapy. Nature.

[5] Yu J, Hu K, Smuga-Otto K, Tian S, Stewart R, Slukvin II, Thomson JA (2009). Human induced pluripotent stem cells free of vector and transgene sequences. Science.

[6] Zhou W, Freed CR (2009). Adenoviral gene delivery can reprogram human fibroblasts to induced pluripotent stem cells. Stem cells.

[7] Stadtfeld M, Nagaya M, Utikal J, Weir G, Hochedlinger K (2008). Induced pluripotent stem cells generated without viral integration. Science.

[8] Okita K, Nakagawa M, Hyenjong H, Ichisaka T, Yamanaka S (2008). Generation of mouse induced pluripotent stem cells without viral vectors. Science.

[9] Si-Tayeb K, Noto FK, Sepac A, Sedlic F, Bosnjak ZJ, Lough JW, Duncan SA (2010). Generation of human induced pluripotent stem cells by simple transient transfection of plasmid DNA encoding reprogramming factors. BMC Dev Biol.

[10] Warren L, Manos PD, Ahfeldt T, Loh YH, Li H, Lau F, Ebina W, Mandal PK, Smith ZD, Meissner A, Daley GQ, Brack AS, Collins JJ, Cowan C, Schlaeger TM, Rossi DJ (2010). Highly efficient reprogramming to pluripotency and directed differentiation of human cells with synthetic modified mRNA. Cell stem cell.

[11] Zhou H, Wu S, Joo JY, Zhu S, Han DW, Lin T, Trauger S, Bien G, Yao S, Zhu Y, Siuzdak G, Scholer HR, Duan L, Ding S (2009). Generation of induced pluripotent stem cells using recombinant proteins. Cell stem cell.

[12] Kim D, Kim CH, Moon JI, Chung YG, Chang MY, Han BS, Ko S, Yang E, Cha KY, Lanza R, Kim KS (2009). Generation of human induced pluripotent stem cells by direct delivery of reprogramming proteins. Cell stem cell.

[13] Cho HJ, Lee CS, Kwon YW, Paek JS, Lee SH, Hur J, Lee EJ, Roh TY, Chu IS, Leem SH, Kim Y, Kang HJ, Park YB, Kim HS (2010). Induction of pluripotent stem cells from adult somatic cells by protein-based reprogramming without genetic manipulation. Blood.

[14] Bosnali M, Edenhofer F (2008). Generation of transducible versions of transcription factors Oct4 and Sox2. Biol Chem.

[15] Lee J, Sayed N, Hunter A, Au KF, Wong WH, Mocarski ES, Pera RR, Yakubov E, Cooke JP (2012). Activation of innate immunity is required for efficient nuclear reprogramming. Cell.

[16] Harreither E, Rydberg HA, Amand HL, Jadhav V, Fliedl L, Benda C, Esteban MA, Pei D, Borth N, Grillari-Voglauer R, Hommerding O, Edenhofer F, Nordén B, Grillari J: **Characterization of a novel cell penetrating peptide derived from human Oct4.** *Cell Regeneration* 2014.,**3**(2) :http://www.cellregenerationjournal.com/content/3/1/2 10.1186/2045-9769-3-2 PMC4230757 25408881

[17] Theunissen TW, Costa Y, Radzisheuskaya A, van Oosten AL, Lavial F, Pain B, Castro LF, Silva JC (2011). Reprogramming capacity of Nanog is functionally conserved in vertebrates and resides in a unique homeodomain. Development.

[18] Aksoy I, Jauch R, Eras V, Bin AC, Chen J, Divakar U, Ng CK, Kolatkar PR, Stanton LW (2013). Sox transcription factors require selective interactions with Oct4 and specific transactivation functions to mediate reprogramming. Stem cells.

[19] Wang Y, Chen J, Hu JL, Wei XX, Qin D, Gao J, Zhang L, Jiang J, Li JS, Liu J, Lai KY, Kuang X, Zhang J, Pei D, Xu GL (2011). Reprogramming of mouse and human somatic cells by high-performance engineered factors. EMBO reports.

[20] Liu X, Sun H, Qi J, Wang L, He S, Liu J, Feng C, Chen C, Li W, Guo Y, Qin D, Pan G, Chen J, Pei D, Zheng H (2013). Sequential introduction of reprogramming factors reveals a time-sensitive requirement for individual factors and a sequential EMT-MET mechanism for optimal reprogramming. Nat Cell Biol.

[21] Thier M, Worsdorfer P, Lakes YB, Gorris R, Herms S, Opitz T, Seiferling D, Quandel T, Hoffmann P, Nothen MM, Brustle O, Edenhofer F (2012). Direct conversion of fibroblasts into stably expandable neural stem cells. Cell stem cell.

[22] Hou P, Li Y, Zhang X, Liu C, Guan J, Li H, Zhao T, Ye J, Yang W, Liu K, Ge J, Xu J, Zhang Q, Zhao Y, Deng H (2013). Pluripotent stem cells induced from mouse somatic cells by small-molecule compounds. Science.

[23] Sugiyama S, Di Nardo AA, Aizawa S, Matsuo I, Volovitch M, Prochiantz A, Hensch TK (2008). Experience-dependent transfer of Otx2 homeoprotein into the visual cortex activates postnatal plasticity. Cell.

